# Perioperative anesthetic considerations and outcomes in Crouzon syndrome: a retrospective analysis of Le Fort III advancement surgery

**DOI:** 10.3389/fped.2026.1840355

**Published:** 2026-05-29

**Authors:** Yue Wei, Lei Wang, Yue Liu, Kunpeng Liu, Lan Yao

**Affiliations:** 1 Department of Anesthesiology, Peking University International Hospital, Beijing, China; 2 Department of Oral and Maxillofacial Surgery, Peking University International Hospital, Beijing, China

**Keywords:** Crouzon syndrome, Le Fort III osteotomy and advancement, operative duration, pediatric anesthesia, postoperative complications

## Abstract

**Objective:**

Le Fort III osteotomy and advancement in children with Crouzon syndrome represents a crucial therapeutic modality for severe midface hypoplasia. These procedures are highly invasive and associated with significant intraoperative hemorrhage and difficult airway management, posing substantial anesthetic risks. This study aims to summarize perioperative anesthetic management strategies, describe perioperative outcomes, and analyze risk factors for postoperative complications in this specific population.

**Methods:**

Medical records of consecutive children with Crouzon syndrome undergoing Le Fort III osteotomy and advancement were retrospectively reviewed from Peking University International Hospital between December 2017 and January 2026. Inclusion criteria were: (i) diagnosis of Crouzon syndrome; (ii) underwent Le Fort III osteotomy and advancement; and (iii) age younger than 18 years at the time of surgery. Data extracted included demographics, perioperative management, complications, fluid and transfusion volumes, and length of stay. Risk factors for postoperative complications were analyzed using univariate and multivariate logistic regression.

**Results:**

A total of 47 children (male, 24) were enrolled, with a median age of 6 years (IQR, 6–10 years). The median operative time was 215 minutes (IQR, 180–279 minutes), intraoperative blood loss was 14.3 mL/kg, and the allogeneic blood transfusion rate was 68.1%. Postoperative complications occurred in 29.8% of patients (14/47). Multivariate logistic regression analysis identified operative duration (odds ratio [OR] = 1.010; 95% confidence interval [CI], 1.001–1.021; *P* = 0.048) as an independent risk factor for postoperative complications. Intraoperative fresh frozen plasma transfusion were not significant in the adjusted model.

**Conclusion:**

Anesthetic management for Le Fort III osteotomy in children with Crouzon syndrome requires meticulous attention to difficult airway anatomy and significant intraoperative hemorrhage. Prolonged operative duration significantly increase the risk of postoperative complications. Future research should prioritize multicenter prospective cohort designs to establish larger, more comprehensive databases capable of validating the present findings.

## Introduction

Crouzon syndrome (CS) is a rare autosomal dominant disorder within the syndromic craniosynostosis spectrum (1), with an estimated prevalence of approximately 1 in 25,000 to 1 in 60,000 live births (2, 3). The premature fusion of cranial sutures restricts normal cranial vault expansion, leading to characteristic craniofacial deformities, increased intracranial pressure, and midfacial hypoplasia, as well as other manifestations such as exophthalmos. Treatment during the preschool or school-age years primarily targets sleep-related breathing disorders, exophthalmos, and orbitofacial deformities secondary to midfacial deficiency, with Le Fort III osteotomy and distraction advancement serving as the fundamental surgical approach (4). However, these operations pose formidable challenges for the anesthesia team. The surgical intervention is highly invasive, frequently associated with significant intraoperative hemorrhage due to extensive subperiosteal dissection and osteotomies near the cranial base. Substantial fluid shifts demand sophisticated hemodynamic monitoring and management (5–8). Additionally, the anatomical constraints of severe midface retrusion, micrognathia, and limited mouth opening create a predictably difficult airway (9, 10).

So far, the existing perioperative evidence for Le Fort III surgery is largely derived from cohorts of mixed syndromic craniosynostosis that aggregate patients with Apert, Pfeiffer, Muenke, and Crouzon syndromes (9, 11–13), yet Crouzon patients were consistently analyzed together with other syndromic subtypes. This aggregation limits the ability to discern syndrome-specific risk profiles, because phenotypic heterogeneity in craniofacial anatomy, airway obstruction patterns, and bleeding tendencies varies considerably across syndromes (11, 14, 15). To date, systematic, procedure-specific data characterizing perioperative anesthetic outcomes during Le Fort III advancement exclusively in CS remain scarce. This evidence gap hinders the development of robust preoperative risk stratification tools and tailored anesthetic protocols for this vulnerable pediatric population.

Therefore, this retrospective study aims to comprehensively evaluate perioperative anesthetic considerations and clinical outcomes in a homogeneous cohort of CS children undergoing Le Fort III advancement surgery. By systematically delineating the incidence of airway difficulties, intraoperative adverse events, blood product utilization, and postoperative complications, we seek to identify pertinent risk factors and inform evidence-based, multidisciplinary perioperative planning, ultimately enhancing the safety and efficacy of anesthetic care for these complex patients.

## Patients and methods

### Study design and patients

This was a retrospective cohort study conducted in accordance with the Declaration of Helsinki and reported following the Strengthening the Reporting of Observational Studies in Epidemiology (STROBE) guideline. The study protocol was approved by the Institutional Review Board (IRB) of Peking University International Hospital (Approval No: 2026-KY-0023-01), and the requirement for individual informed consent was waived due to the retrospective nature of the analysis.

Medical records of consecutive children undergoing Le Fort III osteotomy and advancement for CS at Peking University International Hospital between December 2017 and January 2026 were retrospectively retrieved from our prospectively collected anesthesia database and electronic medical records. Patients were eligible for inclusion if they met the following criteria: (i) genetically or clinically confirmed diagnosis of CS; (ii) underwent primary Le Fort III osteotomy with advancement; and (iii) age younger than 18 years at the time of surgery. Exclusion criteria included: incomplete medical records or missing critical perioperative data.

### Surgical and anesthesia protocols

Preoperative surgical planning is a collaborative effort involving a multidisciplinary team that includes maxillofacial surgeons, a dedicated pediatric anesthesia team, intensivists, neurosurgeons, ear, nose, and throat physicians, and ophthalmologists.

For airway management, video laryngoscopy is typically the primary choice for intubation. Alternative device, such as fiberscope is available for managing both predicted and unpredicted difficult airways. Armored endotracheal tubes are utilized. Anesthesia is maintained with halogenated gas and a continuous infusion of propofol and remifentanil. Intraoperative monitoring encompasses various parameters, including peripheral oxygen saturation, electrocardiography, end-tidal CO_2_, invasive blood pressure through an arterial line, temperature, and diuresis through a urinary catheter. Central venous access is performed based on the anesthesiologist's clinical assessment.

After the surgical procedure, children are admitted to our mixed adult-pediatric ICU to prevent and, if necessary, treat postoperative complications. Extubation typically takes place the day after the surgery.

### Data collection and outcomes

We collected information regarding perioperative management and complications. Demographic data included: gender, height, and weight. Clinical data included: preoperative diagnosis, preoperative comorbidities, surgical procedure, operative duration, postoperative complications, length of hospital stay, and reoperation status. Anesthesia information included: American Society of Anesthesiology (ASA) classification, anesthesia technique, anesthesia monitoring, anesthetic agents, intraoperative fluid balance (input and output), blood product administration, intraoperative vital signs, intraoperative blood gas analysis parameters, and occurrence of intraoperative adverse events. Laboratory examinations included: hemoglobin (Hb), hematocrit (Hct), arterial blood gas analysis (lactate, base excess, pH), and electrolytes (potassium, ionized calcium) and other relevant indicators. The airway was considered difficult if the Cormack-Lehane (CL) airway grade was ≥ 3. Hypothermia was defined as a rectal temperature below 36°C throughout the surgical procedure. Hypotension was defined as systolic blood pressure (SBP) more than 2 standard deviations below the sex- and age-specific reference mean (approximately the 2.5th percentile) (16).

Data were primarily extracted from the Electronic Medical Record system and the electronic anesthesia information system. All data were independently entered by two anesthesiologists to ensure accuracy and reliability.

The primary outcome was the rate of postoperative complications occurring within 30 days after surgery. Postoperative complications were recorded using standardized diagnostic criteria as follows:.

Cerebrospinal fluid (CSF) leak was defined as frank drainage of clear fluid from the wound, nose, or ear with positive glucose test strip (> 30 mg/dL).

Carotid-cavernous fistula was defined as the presence of at least two clinical features (pulsatile proptosis, chemosis, cranial bruit, or cranial nerve palsy) confirmed by digital subtraction angiography.

Hyponatremia was defined as serum sodium < 130 mmol/L on two separate measurements, accompanied by clinical symptoms (altered consciousness, seizure, or vomiting), after excluding pseudohyponatremia.

Surgical site infection was defined according to CDC criteria as purulent wound drainage with positive culture, or deep infection requiring surgical intervention, within 30 days postoperatively.

Wound hemorrhage was defined as postoperative bleeding requiring transfusion (≥ 1 U PRBC), bedside surgical hemostasis, or return to operating room for exploration, excluding normal postoperative serosanguinous drainage.

Pneumonia was defined as new-onset pulmonary infiltrate on chest imaging plus at least two of: fever > 38°C, leukocytosis or leukopenia, purulent sputum, or worsening oxygenation, occurring > 48 hours postoperatively.

The validated Clavien-Dindo surgical classification system based on severity of complication was also applied (17, 18).

Grade I: Adverse event that alters the standard postoperative course without requiring a specific treatment.
Grade II: Pharmacologic treatment or minor intervention required.
Grade III: Requiring surgical, endoscopic, or radiological intervention.
  - Grade IIIa: Intervention not under general anesthesia.
  - Grade IIIb: Intervention under general anesthesia.
Grade IV: Life-threatening complication requiring ICU management.
  - Grade IVa: Single-organ dysfunction.
  - Grade IVb: Multi-organ dysfunction.
Grade V: Death.

Secondary outcomes included: (i) intraoperative blood loss (estimated from suction canister volume and surgical sponge weighing); (ii) blood product transfusion requirements (packed red blood cells [PRBC], fresh frozen plasma [FFP], platelets); (iii) duration of surgery and anesthesia; (iv) postoperative ICU admission and duration of mechanical ventilation; and (v) hospital length of stay.

### Statistics

Given the retrospective study design and the rarity of CS, formal *a priori* sample size calculation was not feasible. Instead, we employed consecutive enrollment including all eligible children who underwent Le Fort III osteotomy and advancement for CS at our institution during the 8-year study period.

Categorical variables were summarized as frequencies and percentages, and continuous variables as median with interquartile range [IQR] or mean ± standard deviation (SD) depending on normality of distribution (assessed using the Shapiro–Wilk test). Patient characteristics, anesthetic management details, and outcomes were compared between groups with and without complications using the Chi-square test or Fisher's exact test for categorical variables, and the Student's t-test or Mann–Whitney U test for continuous variables as appropriate. Univariate logistic regression was performed to identify potential risk factors associated with postoperative complications. Variables with a *P*-value < 0.1 in univariate analysis were subsequently entered into a multivariate logistic regression model using backward elimination to identify independent risk factors. Results were reported as odds ratios (OR) with 95% confidence intervals (CI) and *P*-values. Statistical significance was defined as a two-tailed *P*-value < 0.05. All statistical analyses were performed using SPSS version 19.0.

## Results

### Patient characteristics

A total of 47 children (24 males and 23 females) were enrolled in this study. The median age at surgery was 6 years (interquartile range [IQR], 6–10 years), with a median weight of 22.5 kg (IQR, 20.0–35.0 kg) and height of 120.0 cm (IQR, 115.0–140.0 cm). Preoperative assessment revealed that most patients (93.6%, 44/47) had obstructive sleep apnea (OSA). Regarding physical status classification, 38.3% (18/47) were classified as ASA II and 61.7% (29/47) as ASA III (Table 1).

**Table 1:** Characteristics of all patients.

Variables	*N*	% or value
Total	47	
Age, median (IQR), y		6 (6, 10)
Height, median (IQR), cm		120.0 (115.0, 140.0)
Weight, median (IQR), kg		22.5 (20.0, 35.0)
Sex		
Male	24	51.1
Female	23	48.9
ASA physical status		
II	18	38.3
III	29	61.7
Preoperative comorbidities		
Obstructive sleep apnea	44	93.6
Hydrocephalus	8	17.0
Spine abnormalities	4	8.5
Strabismus	20	42.6
Arnold- Chiari malformation	21	44.7

### Anesthesia management

All patients successfully underwent Le Fort III osteotomy and advancement under general anesthesia. The median operative time was 215.0 minutes (IQR, 180.0–279.0 minutes), and the median anesthesia duration was 280.0 minutes (IQR, 240.0–334.0 minutes). The median intraoperative blood loss was 14.3 mL/kg (IQR, 9.5–21.7 mL/kg); only one patient (2.1%) experienced blood loss exceeding 50 mL/kg. Blood product transfusion was administered to 68.1% (32/47) of patients. The median red blood cell transfusion was 13.1 mL/kg (IQR, 0-17.0 mL/kg), with 29.8% (14/47) receiving fresh frozen plasma (median 0 mL/kg, IQR 0-6.1 mL/kg). No patients required platelet transfusion (Table 2).

**Table 2:** Perioperative variables and operative outcomes.

Variables	*n*	%	Value
Total patients	47		
Operative duration, median (IQR), min			215.0 (180.0, 279.0)
Anesthesia duration, median (IQR), min			280.0 (240.0, 334.0)
Intraoperative blood loss, median (IQR), mL/kg			14.3 (9.5, 21.7)
Patients requiring blood transfusion	32	68.1	
Packed red blood cells, median (IQR), mL/kg			13.1 (0, 17.0)
Fresh frozen plasma, median (IQR), mL/kg			0 (0, 6.1)
Platelet transfusion	0	0	
Crystalloid, median (IQR), mL/kg			42.3 (25.8, 52.8)
Colloid fluids, median (IQR), mL/kg			0 (0, 9.5)
Albumin, median (IQR), g			10.0 (0, 30.0)
Tranexamic acid, median (IQR), mg/kg			0 (0, 19.5)
Airway management			
Difficult intubation (Cormack ≥ III)	4	8.5	
Fiber-optic guided intubation	3	6.4	
Video laryngoscopy intubation	44	93.6	
Intraoperative monitoring			
Invasive arterial catheter	47	100	
Central venous catheter	16	34.0	
Intraoperative complications			
Blood loss > 50 mL/kg	1	2.1	
Hypothermia	20	42.6	
Hypotension	14	29.8	
Mechanical ventilation duration median (IQR), h			21.0 (18.0, 22.0)
Time of extubation in cases of postoperative tracheal intubation			
< 24 h postoperatively	42	89.4	
> 24 h postoperatively	5	10.6	
ICU admission postoperatively, median (IQR), days			2.0 (2.0, 2.0)
Duration of admission, median (IQR), days			21.0 (18.0, 26.0)

Intraoperative fluid management consisted predominantly of crystalloid administration, with a median volume of 42.3 mL/kg (IQR, 25.8-52.8 mL/kg). Colloid fluids were administered to selected patients (median 0 mL/kg, IQR 0- 9.5 mL/kg), and albumin was given at a median dose of 10.0 g (IQR, 0–30.0 g). Pharmacologic interventions for blood conservation included tranexamic acid, which was administered at the discretion of the anesthesiologist (median dose 0 mg/kg, IQR 0–19.5 mg/kg), indicating variable utilization across cases (Table 2).

Regarding airway management, 93.6% (44/47) of patients underwent video laryngoscopy-assisted intubation, while 6.4% (3/47) required fiber-optic bronchoscopy guidance due to anticipated difficult airway anatomy. All patients (100%) received invasive arterial catheterization for hemodynamic monitoring, and 34.0% (16/47) had central venous catheterization. Intraoperative hypothermia occurred in 42.6% (20/47) of patients, and hypotension in 29.8% (14/47) (Table 2).

### Postoperative complications

Postoperative complications occurred in 29.8% (14/47) of patients within the 30-day observation window. According to the Clavien-Dindo classification, Grade II complications (requiring pharmacologic treatment or blood transfusion without surgical intervention) were observed in 8 patients (17.0%), including wound hemorrhage managed conservatively (*n* = 4, 8.5%), cerebrospinal fluid leak (*n* = 2, 4.3%), pneumonia (*n* = 1, 2.1%), and hyponatremia (*n* = 1, 2.1%). Grade IIIb complications (requiring surgical intervention under general anesthesia) occurred in 6 patients (12.8%), comprising wound hemorrhage necessitating reoperation (*n* = 2, 4.3%), accidental fracture (*n* = 2, 4.3%), wound infection (*n* = 1, 2.1%), and carotid-cavernous fistula (*n* = 1, 2.1%). No Grade I, IIIa, IV, or V complications were recorded, and no perioperative mortality occurred (Table 3).

**Table 3:** Postoperative complications overall.

Postoperative complication	*n*	%	Clavien-Dindo grade
Pneumonia	1	2.1	II
Cerebrospinal fluid leak	2	4.3	II
Hyponatremia	1	2.1	II
Wound hemorrhage	4	8.5	II
	2	4.3	IIIb
Wound infection	1	2.1	IIIb
Accidental fracture	2	4.3	IIIb
Carotid-cavernous fistula	1	2.1	IIIb
Total with any complication	**14**	**29.8**	

Bold values indicate the total number and overall percentage of patients who experienced at least one postoperative complication.

### Risk factor analysis for postoperative complications

Univariate logistic regression analysis identified operative duration (*P* = 0.014), and fresh frozen plasma transfusion (*P* = 0.077) as potential risk factors associated with postoperative complications (*P* < 0.1) (Table 4). However, multivariate logistic regression analysis revealed that only operative duration was an independent risk factor for postoperative complications (odds ratio [OR] = 1.010; 95% confidence interval [CI], 1.001–1.021; *P* = 0.048) (Table 5).

**Table 4:** Univariate analysis of factors associated with postoperative complications.

Variables	Postoperative complications		t/Z/*χ*2 -value	*p*-value
	Yes (*n* = 14)	No (*n* = 33)		
Age, median (IQR), y	6.5 (5.0, 9.3)	6.0 (6.0, 11.0)	−0.736	0.462
Male sex, *n* (%)	9 (64.3)	15 (45.5)	1.395	0.238
BMI, median (IQR), kg/m^2^	16.1 (14.6, 17.6)	15.7 (14.2, 20.5)	−0.186	0.852
ASA III, *n* (%)	7 (50.0)	22 (66.7)	1.155	0.282
Severe OSA, *n* (%)	10 (71.4)	20 (60.6)	0.499	0.480
Intracranial hypertension, *n* (%)	1 (7.1)	7 (21.2)	0.562	0.454
Preoperative Hb, mean ± SD, g/dL	124.7 ± 11.1	128.8 ± 11.2	−1.155	0.254
Operative duration, median (IQR), min	246.5 (220.3, 301.3)	210.0 (160.0, 258.5)	−2.467	0.014
Difficult airway, *n* (%)	1 (7.1)	3 (9.1)		1.000
Estimated blood loss, median (IQR), mL/kg	13.8 (11.8, 19.1)	14.6 (9.1, 23.6)	−0.151	0.880
PRBC transfusion, median (IQR), mL/kg	10.0 (0, 15.5)	14.8 (0, 17.0)	−1.006	0.314
FFP transfusion, median (IQR), mL/kg	0 (0, 0)	0 (0, 0)	−1.770	0.077
Crystalloid, median (IQR), mL/kg	43.2 (29.5, 55.1)	36.1 (22.7, 51.5)	−0.756	0.450
Colloid, median (IQR), mL/kg	0 (0, 10.9)	0 (0, 9.1)	−0.189	0.850
Urine output, median (IQR), mL/kg	15.8 (8.6, 19.3)	16.0 (6.6, 28.3)	−0.558	0.577
Tranexamic acid use, *n* (%)	2 (14.3)	14 (42.4)	2.326	0.127
Hypothermia, *n* (%)	8 (57.1)	12 (36,4)	1.736	0.188
Hypotensionn, (%)	5 (35.7)	9 (27.3)	0.053	0.818

Continuous variables were compared using Student's t-test (normally distributed) or Mann–Whitney U test (non-normally distributed); categorical variables were compared using *χ*^2^ test or Fisher's exact test. Hb, hemoglobin; PRBC, packed red blood cells; FFP, fresh frozen plasma; ASA, American Society of Anesthesiology; OSA, obstructive sleep apnea; BMI, body mass index.

**Table 5:** Multivariate analysis of factors associated with postoperative complications.

Variable	*n*	Events	OR (95% CI)	*P*-value
Operative duration (per min)	47	14	1.010 (1.001–1.021)	0.048
FFP transfusion (per mL/kg)	47	14	0.816 (0.649–1.027)	0.083

FFP, fresh frozen plasma; OR, odds ratio; CI, confidence interval.

## Discussion

Our series of 47 children with CS undergoing Le Fort III osteotomy and advancement represents one of the largest cohorts published to date. The overall postoperative complication rate was 29.8% (14/47), with 12.8% (6/47) of patients experiencing severe complications (Clavien-Dindo grade ≥ 3a) (17). These rates are similar to, or somewhat lower than, those reported in previously published studies of mixed syndromic craniosynostosis cohorts (18–20). These findings indicate that despite advances in distraction osteogenesis techniques, major craniofacial osteotomies in this patient population remain associated with substantial risks of complications.

Multivariate regression analysis confirmed that operative duration was an independent predictor of postoperative complications (OR = 1.010 per minute). We interpret this finding cautiously, recognizing that operative duration in Le Fort III surgery likely functions as a composite surrogate marker of underlying case complexity, such as severe craniofacial deformity, limited surgical access secondary to midface retrusion, the need for concomitant calvarial bone grafting, and intraoperative troubleshooting such as distraction vector adjustment. Simultaneously, it serves as a source of cumulative exposure to time-dependent hazards (21). This dual interpretation aligns with the findings of Cheng et al.'s systematic review and meta-analysis of 66 observational studies across surgical specialties, which demonstrated a robust association between prolonged operative time and complications across surgical specialties (22). Notably, Cheng et al. acknowledge that longer operative times often indicate more complex or difficult surgeries, yet emphasize that the time-complication association persists even after adjustment for patient comorbidities and procedural complexity, suggesting that operative duration captures both confounded and independent risk dimensions.

Beyond its role as a complexity proxy, prolonged operative time introduces hazards that are particularly relevant to Le Fort III osteotomy. These procedures involve extensive subperiosteal dissection, multiplanar osteotomies traversing the cranial base and pterygomaxillary region, and manipulation within millimeters of the dura mater and venous sinuses. As operative time extends, cumulative exposure increases the risk of occult dural injury with subsequent cerebrospinal fluid leak, venous air embolism through exposed diploic or dural venous channels, and progressive hemorrhage from abnormal vascular anatomy (23). We acknowledge that physiological disturbances such as hypothermia, anesthetic drug accumulation, and coagulopathy are common to all prolonged surgical procedures across diverse specialties (24, 25); however, in Le Fort III surgery, these universal time-dependent risks are superimposed upon anatomy-specific hazards, creating a amplified “time-risk” gradient that warrants procedure-specific vigilance.

Of note, intraoperative FFP transfusion (*P* = 0.083) in this study demonstrated marginal associations with postoperative complications in univariate analysis (*P* < 0.1), yet failed to maintain independent significance in the multivariate model after adjusting for operative duration. Although this factor did not achieve statistical significance as independent risk factors, it remains important mediating indicator for perioperative quality improvement. Extended time on the operating table can gradually worsen anemia, disrupt the body's natural clotting rhythm. The apparent “protective” effect of FFP transfusion likely reflects confounding by indication—sicker patients may have received more FFP (26). Therefore, perioperative management should emphasize optimization of vigilant intraoperative coagulation monitoring and management.

In this study, 68.1% of children received allogeneic blood transfusion, slightly lower than the 82% reported by Glover et al. (11) in their mixed syndromic craniosynostosis cohort undergoing Le Fort III surgery. This lower transfusion rate may be partly attributable to our exclusive inclusion of CS patients, whereas the cohort of Glover et al. comprised various subtypes of syndromic craniosynostosis. Notably, certain subtypes—particularly Apert syndrome—are associated with substantially greater intraoperative blood loss (20), which may have elevated the overall transfusion requirement in their mixed cohort. The median transfusion volume in our study [14.3 (9.5, 21.7) mL/kg] was intermediate between that reported by Glover et al. (17 ± 15 mL/kg) (11) and that documented by Restivo et al. (12.6 ± 16 mL/kg) (12). A review by Ma et al. (27) emphasized the pivotal role of perioperative blood management in craniosynostosis repair, encompassing preoperative erythropoietin administration, intraoperative tranexamic acid (TXA) use, and cell salvage techniques. Although TXA utilization varied considerably in this study (median dosage 0 mg/kg, IQR 0–19.5 mg/kg), its efficacy as an effective strategy to reduce perioperative transfusion requirements has been validated by multiple studies (28, 29). Optimizing surgical protocols, applying antifibrinolytic agents, and implementing restrictive transfusion strategies to minimize transfusion demands constitute crucial steps toward improving outcomes in this pediatric population.

CS is frequently associated with midfacial hypoplasia, nasopharyngeal stenosis, tracheal cartilage abnormalities, and cervical spine anomalies, rendering airway management one of the most high-risk aspects of the perioperative period. In this study, all children in our study underwent successful endotracheal intubation. We encountered difficult airways in 7.5% of cases, which appears notably lower than the roughly 14% incidence reported by Glover et al. (11) among children with syndromic craniosynostosis. This discrepancy may be attributed to our center's routine use of video laryngoscopy for intubation. Although Cuperus et al. demonstrated that immediate extubation is feasible following midface advancement in patients with syndromic craniosynostosis (9, 13), the majority of studies continue to advocate for a delayed extubation strategy (11, 12). In the present study, 94.3% of patients had concurrent obstructive sleep apnea (OSA). We implemented a carefully staged extubation protocol for this cohort. All patients went directly to the ICU postoperatively, and our team ensured everyone received head and facial CT scans within the first eight hours. This allowed us to exclude severe complications like anterior cranial base fractures, intracranial hemorrhage, or retrobulbar hematoma before removing the tube. Extubation happened only once we had confirmed airway patency and overall stability.

This study has several limitations. First, its retrospective, single-center design and limited sample size (*n* = 47) preclude causal inference and may have constrained our ability to detect modest but clinically relevant associations; therefore, our findings should be interpreted as exploratory and hypothesis-generating rather than confirmatory. Second, we recognize that operative duration likely functions as a composite surrogate marker for unmeasured confounders such as intrinsic case complexity, severity of craniofacial deformity, and team experience, rather than as a purely independent causal variable. Third, we did not have granular data on specific intraoperative phases such as osteotomy, advancement, or hemostasis, which would have allowed us to weigh their relative contributions to complications. Future research should prioritize multicenter prospective cohort designs to establish larger, more comprehensive databases capable of validating the present findings.

Our findings carry some implications for clinical practice. Operative time represents a modifiable perioperative variable that should become a focal point for quality improvement initiatives. Careful preoperative planning paired with genuine multidisciplinary teamwork can meaningfully shorten surgical time. When experienced surgeons and anesthesiologists collaborate closely from the outset, patients ultimately benefit from smoother procedures and improved outcomes.

## Conclusion

Le Fort III midface advancement via distraction osteogenesis for CS represents a technically demanding, high-risk major surgical procedure. This study confirms operative time as an independent predictor of postoperative complications. Implementation of streamlined surgical workflows to minimize operative time through multidisciplinary collaboration within high-volume craniofacial centers, coupled with rigorous adherence to blood conservation protocols, constitutes the critical strategy for improving perioperative outcomes in this complex pediatric population.

## Data Availability

The raw data supporting the conclusions of this article will be made available by the authors, without undue reservation.

## References

[1] Hwang S-K Park K-S Park S-H Hwang SK. Update of diagnostic evaluation of craniosynostosis with a focus on pediatric systematic evaluation and genetic studies. J Korean Neurosurg Soc. (2016) 59(3):214–18. 10.3340/jkns.2016.59.3.214 27226851 PMC4877542

[2] Cohen MM Jr Krelborg S. Birth prevalence studies of the Crouzon syndrome: comparison of direct and indirect methods. Clin Genet. (1992) 41(1):12–15. 10.1111/j.1399-0004.1992.tb03620.x 1633640

[3] Di Rocco F Arnaud E Renier D. Evolution in the frequency of nonsyndromic craniosynostosis. J Neurosurg Pediatr. (2009) 4(1):21–25. 10.3171/2009.3.PEDS08355 19569905

[4] Raffaini M Magri AS Genitori L Arcuri F. Algorithm to the treatment of Crouzon syndrome. J Craniomaxillofac Surg. (2022) 50(2):124–33. 10.1016/j.jcms.2021.11.008 34857441

[5] Thomas K Hughes C Johnson D Das S. Anesthesia for surgery related to craniosynostosis: a review. Part 1. Pediatric Anesthesia. (2012) 22(11):1033–41. 10.1111/j.1460-9592.2012.03927.x 22928738

[6] Hughes C Thomas K Johnson D Das S. Anesthesia for surgery related to craniosynostosis: a review. Part 2. Pediatric Anesthesia. (2013) 23(1):22–27. 10.1111/j.1460-9592.2012.03922.x 22882624

[7] Pearson A Matava C. Anaesthetic management for craniosynostosis repair in children. BJA Educ. (2016) 16(12):410–16. 10.1093/bjaed/mkw023

[8] Boudjenah B Adham AMB Chinnappa SM Assaf O Al–Khelaifi M. “An Approach to the Airway Management in Children with Craniofacial Anomalies”. In: Shallik NA, editor. Special Considerations in Human Airway Management. London: IntechOpen (2021). p. 1–28. 10.5772/intechopen.93426

[9] Cuperus IE Bernard SE Joosten KF Wolvius EB Pullens B Mathijssen IM. Perioperative airway management for midface surgery in children with syndromic craniosynostosis; a single center experience with immediate extubation. Pediatric Anesthesia. (2025) 35(5):380–88. 10.1111/pan.15086 39982051 PMC11975210

[10] Garcia-Marcinkiewicz AG Stricker PA. Craniofacial surgery and specific airway problems. Pediatric Anesthesia. (2020) 30(3):296–303. 10.1111/pan.13790 31837242

[11] Glover CD Fernandez AM Huang H Derderian C Binstock W Reid R. Perioperative outcomes and management in midface advancement surgery: a multicenter observational descriptive study from the pediatric craniofacial collaborative group. Pediatric Anesthesia. (2018) 28(8):710–18. 10.1111/pan.13418 29920872

[12] Restivo A Rondelli E Giani M Moretti M Fossati C Rona R. Perioperative care for children with syndromic craniofacial synostosis undergoing Le Fort III surgery: a retrospective cohort study. J Craniofac Surg. (2024) 35(6):1692–95. 10.1097/SCS.0000000000010400 38856193 PMC11346705

[13] Cuperus IE Mathijssen IM van Veelen M-LC Bouzariouh A Stubelius I Kölby L. A European multicenter outcome study of perioperative airway management policies following midface surgery in syndromic craniosynostosis. Plast Reconstr Surg. (2024) 154(6):1281–92. 10.1097/PRS.0000000000011317 38289904 PMC11584185

[14] Alam MK Alfawzan AA Abutayyem H Kanwal B Alswairki HJ Verma S. Craniofacial characteristics in Crouzon’s syndrome: a systematic review and meta-analysis. Sci Prog. (2023) 106(1):00368504231156297. 10.1177/00368504231156297 36803068 PMC10358517

[15] Tiberio F Polito L Salvati M Di Pietro L Massimi L Parolini O. Current understanding of Crouzon syndrome pathophysiology and new therapeutic approaches. J Craniofac Surg. (2025) 36(8):2959–70. 10.1097/SCS.0000000000011376 40227035

[16] De Graaff JC Pasma W Van Buuren S Duijghuisen JJ Nafiu OO Kheterpal S. Reference values for noninvasive blood pressure in children during anesthesia: a multicentered retrospective observational cohort study. Anesthesiology. (2016) 125(5):904. 10.1097/ALN.0000000000001310 27606930 PMC5259808

[17] Dindo D Demartines N Clavien P-A. Classification of surgical complications: a new proposal with evaluation in a cohort of 6336 patients and results of a survey. Ann Surg. (2004) 240(2):205–13. 10.1097/01.sla.0000133083.54934.ae 15273542 PMC1360123

[18] Goldstein JA Thomas Paliga J Taylor JA Bartlett SP. Complications in 54 frontofacial distraction procedures in patients with syndromic craniosynostosis. J Craniofac Surg. (2015) 26(1):124–28. 10.1097/SCS.0000000000001320 25569391

[19] Raposo-Amaral CE Denadai R Pereira-Filho JC Vieira PH Ghizoni E Raposo-Amaral CA. Serious complications after Le Fort III distraction osteogenesis in syndromic craniosynostosis: evolution of preventive and therapeutic strategies. J Craniofac Surg. (2018) 29(6):1397–1401. 10.1097/SCS.0000000000004770 29944568

[20] Purnell CA Evans M Massenburg BB Kim S Preston K Kapadia H. Lefort II distraction with zygomatic repositioning versus Lefort III distraction: a comparison of surgical outcomes and complications. J Craniomaxillofac Surg. (2021) 49(10):905–13. 10.1016/j.jcms.2021.03.003 33965326

[21] Al-Namnam N Hariri F Rahman Z. Distraction osteogenesis in the surgical management of syndromic craniosynostosis: a comprehensive review of published papers. Br J Oral Maxillofac Surg. (2018) 56(5):353–66. 10.1016/j.bjoms.2018.03.002 29661509

[22] Cheng H Clymer JW Chen BP-H Sadeghirad B Ferko NC Cameron CG. Prolonged operative duration is associated with complications: a systematic review and meta-analysis. J Surg Res. (2018) 229:134–44. 10.1016/j.jss.2018.03.022 29936980

[23] Dunaway DJ Britto JA Abela C Evans RD Jeelani NO. Complications of frontofacial advancement. Childs Nerv Syst. (2012) 28(9):1571–76. 10.1007/s00381-012-1804-y 22872275

[24] Yin L Wang H Yin X Hu X. Impact of intraoperative hypothermia on the recovery period of anesthesia in elderly patients undergoing abdominal surgery. BMC Anesthesiol. (2024) 24(1):124. 10.1186/s12871-024-02509-6 38561683 PMC10983640

[25] Chocron Y Azzi AJ Galli R Alnaif N Atkinson J Dudley R. Operative time as the predominant risk factor for transfusion requirements in nonsyndromic craniosynostosis repair. Plast Reconstr Surg Glob Open. (2020) 8(1):e2592. 10.1097/GOX.0000000000002592 32095402 PMC7015599

[26] Stricker PA Shaw TL Desouza DG Hernandez SV Bartlett SP Friedman DF. Blood loss, replacement, and associated morbidity in infants and children undergoing craniofacial surgery. Pediatric Anesthesia. (2010) 20(2):150–59. 10.1111/j.1460-9592.2009.03227.x 20078812

[27] Ma B Sepsick H Scott C Deek A. Perioperative management for craniosynostosis repair in a pediatric population: a narrative review. J Oral Maxillofac Anesth. (2025) 4:17. 10.21037/joma-25-12

[28] Manasyan A Roohani I Wolfe E Turk M Urata MM Hammoudeh JA. Tranexamic acid is associated with reduced blood loss and transfusion requirement in pediatric midface reconstruction. J Oral Maxillofac Surg. (2025) 83(3):286–93. 10.1016/j.joms.2024.10.021 39581566

[29] Dadure C Sauter M Bringuier S Bigorre M Raux O Rochette A. Intraoperative tranexamic acid reduces blood transfusion in children undergoing craniosynostosis surgery: a randomized double-blind study. Surv Anesthesiol. (2011) 55(6):293–4. 10.1097/01.SA.0000407024.78528.c8 21358317

